# Low back pain and work-related factors among home health care workers with self-governing or conventional team structure – a natural experiment with a cross-sectional design

**DOI:** 10.1007/s00420-025-02134-x

**Published:** 2025-03-17

**Authors:** Kathrine Greby Schmidt, Laura Grace Downs Tuck, Anders Bruun Nielsen, Charlotte Diana Nørregaard Rasmussen

**Affiliations:** 1 The National Research Centre for the Working Environment, Copenhagen, 2100 Denmark; 2 Department of Sports Science and Clinical Biomechanics, University of Southern Denmark, Odense, Denmark

**Keywords:** Home health care, Self-governing teams, LBP, Natural experiment

## Abstract

**Objectives:**

Compare home health care (HHC) with self-governing and conventional team structure regarding self-reported low back pain (LBP) and work-related factors.

**Methods:**

A natural experiment was assessed using a cross-sectional design. Primary outcome was LBP intensity; secondary outcomes included LBP duration and work limitations as well as intensity, duration and work limitations of neck/shoulder pain, stress, productivity, influence at work, meaning at work, sickness absence, interpersonal collaboration, and variation in physical behaviour. Home health care (HHC) workers in the self-governing teams were surveyed about their appraisal of the self-governing structure. Data were collected through a questionnaire, except for physical behaviour, which was obtained via accelerometry. Differences between groups were analyzed using t-tests.

**Results:**

From 10 HHC-teams across four municipalities, 125 HHC-workers completed the questionnaire (self-governing *n* = 60; conventional *n* = 65). LBP intensity was similar among HHC-workers in the two team structures (self-governing = 4.1; conventional = 4.0, 0–10 scale). Self-governing teams experienced significantly higher levels of (i) meaning at work (5.8 points, 0-100 scale), (ii) improved collaboration with manager (7.5 points, 0-100 scale) and (iii) improved collaboration with needs assessors (11.9 points, 0-100 scale) compared to conventional teams. No significant differences were found in the other outcomes.

**Conclusions:**

The higher scores for self-governing teams in meaning at work, collaboration with manager and collaboration with needs assessors are positive. The lack of a lower report in LBP and neck/shoulder pain calls for more focused efforts to enhance HHC-workers’ health in addition to the reorganization into the self-governing structure.

## Introduction

The demographic development of a growing aging population and more people living with chronic health conditions poses a challenge for the welfare state (Hussain et al. 2012). The increasing healthcare needs predict a future high demand for home health care (HHC) workers. Recruitment of HHC-workers has proven to be difficult (Danish Agency for Labour Marked and Recruitment 2023), indicating that initiatives that make work more healthy and sustainable for HHC-workers are essential to improving workforce retention and potentially recruitment over time (OECD 2020).

HHC-workers find their work to be physically demanding and exhausting (Albanesi et al. 2022; Delp et al. 2010; Grasmo et al. 2021; Merkus et al. 2019), they experience a high prevalence of musculoskeletal pain (Andersen et al. 2012; Rasmussen et al. 2019) and a diminished health status (Jensen et al. 2019). The combination of these aspects may explain the high rates of sickness absence and early retirement among HHC-workers (Andersen et al. 2012). Until now, several workplace health initiatives have fallen short in effectively addressing work-related musculoskeletal pain among HHC-workers (Albanesi et al. 2022; Freiberg et al. 2016).

The current structure in Danish HHC utilize a designated scheduling coordinator who is typically responsible for managing the distribution of citizens among HHC-workers. To the best of their abilities, the scheduling coordinator tries to consider a multitude of factors such as the relationship between HHC-workers and citizens, time-sensitive appointments, and daily changes arising from the evolving care situations of the citizens (Rambøll 2018). This conventional approach might not be the best at distributing the workload as indicated by a study conducted in Norway, which objectively measured physical work demands in 13 HHC-units, showing an uneven distribution of work tasks among the HHC-workers (Tjøsvoll et al. 2022). Furthermore, the physical work required to care for a citizen is an important predictor for end-of-day pain (Czuba et al. 2012) and physical exertion (Jakobsen et al. 2022). Thus, knowledge about alternative ways to plan and organize the visits and the care for the citizens to reduce high work demands for HHC-workers is needed.

From 2022 to 2023, the Danish National Board of Social Services funded projects that aimed to develop and test new approaches to build greater sustainability in HHC (Buch and Topholm 2023). Drawing inspiration from the Dutch concept Buurtzorg (Hegedüs et al. 2022) and the Swedish concept Västervik (Schou 2022), HHC-teams within 25 municipalities restructured their HHC into small self-governing teams (Buck, M.S., Topholm, E.H., Christensen, J., 2024). The restructuring of HHC into smaller self-governing teams could potentially result in a more evenly distributed workload than the conventional structure and thereby address work-related musculoskeletal pain. However, this remains to be established. A previous study found that eldercare workers in nursing home wards are more likely to experience LBP the higher the number of residents they care for over a year. In contrast, self-managed scheduling in HHC-teams may promote a more balanced distribution of work tasks. As a result, restructuring HHC-workers into smaller teams could be a beneficial organizational approach to prevent and reduce LBP among HHC-workers.

Self-governing teams have only been implemented in a small number of HHC-teams across municipalities in Denmark and has not been compared to conventional teams regarding LBP and work-related factors. The present study will assess a natural experiment with self-governing team structure (Craig et al. 2012, 2022) using a cross-sectional design (Leatherdale 2019). The opportunity of gaining knowledge from a natural experiment arises when events or interventions occur without being planned as a research project (Craig et al. 2012). The present natural experiment, where some HHC-workplaces have transitioned to self-government while other HHC-teams have not, offers a unique opportunity to compare the working environment between the two team structures. The use of a cross-sectional design provides point prevalence data, offering valuable insights that can be used to identify areas where future interventions should be targeted to improve the work environment for HHC-workers within the self-governing structure.

Thus, the aim of this study was to compare HHC with self-governing and conventional team structure regarding self-reported LBP and work-related factors using a natural experiment with a cross-sectional design.

## Methods

We assessed a natural experiment using a cross-sectional design. The study did not fall under the definition of the laws defined in Committee Act § 2 and § 1, and could be initiated without approval from The Committees on Health Research Ethics for the Capital Region of Denmark (Ref number: F-23031959). The study was registered as a clinical trial with ISRCTN (Ref number: 13586262) prior to data collection. Participants were given written information about the study and gave their consent prior to enrollment.

### Recruitment and inclusion criteria

A compilation sourced from the Danish National Board of Social Services, detailed the Danish municipalities that had received funds to implement self-governing teams in HHC in 2022–2023 (Buch and Topholm 2023). Based on this we initiated contact by email with municipalities that had undergone the restructure to self-governing teams (i.e. self-governing teams) as well as teams that have not yet undergone the restructure to self-governing teams (i.e. conventional teams).

Eligibility criteria for a conventional team was: (i) working day shifts and (ii) not yet undergone the restructure to self-governing teams. Eligibility criteria for a self-governing team was: (i) divided in groups with a maximum of 12 HHC-workers, (ii) working day shifts and (iii) having worked as self-governing for a minimum of six months. All HHC-workers within the participating teams were invited to participate.

### Conventional structured teams and teams structured as self-governing

**Fig. 1:**
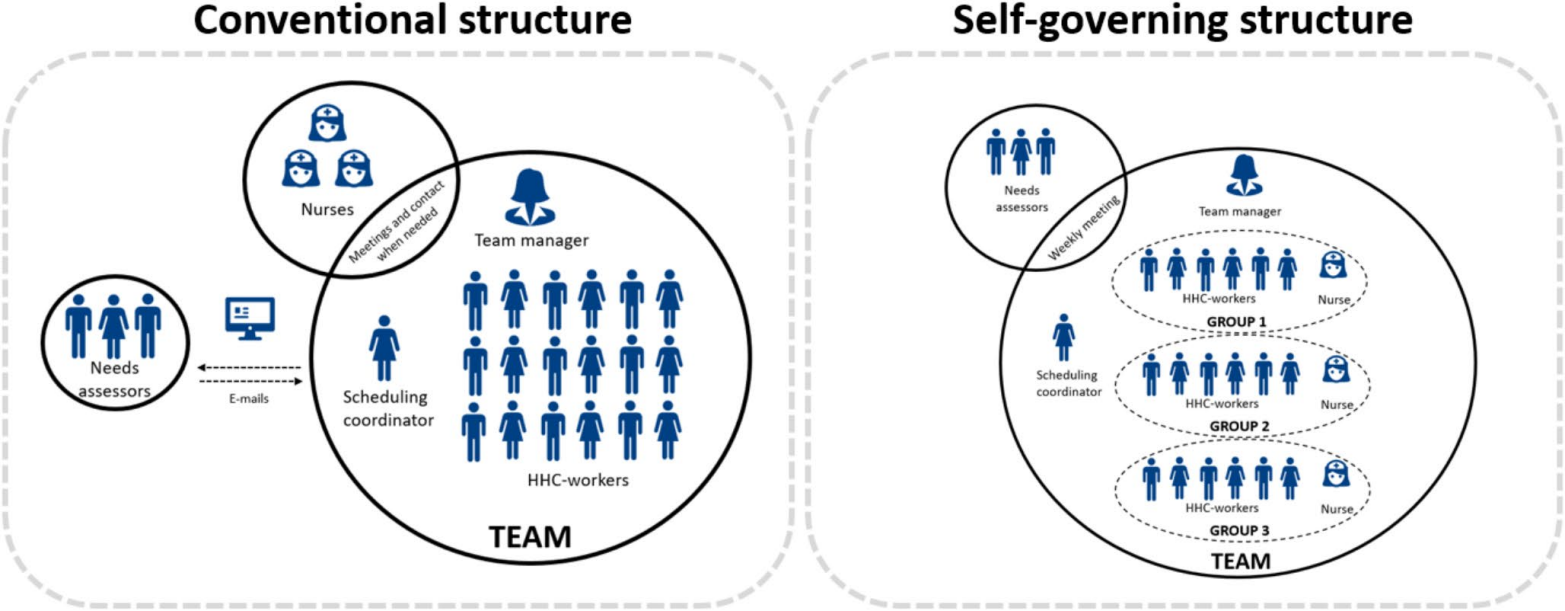
The structure of conventional teams versus self-governing teams included in this study

There is a variation among the funded municipalities in their understanding and implementation of the self-governing elements, including team size and degree of interdisciplinary integration (Buck, M.S., Topholm, E.H., Christensen, J., 2024). However, all self-governing teams included in this study have organized their teams the same way. Figure 1 illustrates the organisational differences between the conventional and self-governing teams included in this study. In conventional teams, there is typically one team manager responsible for 20–30 HHC-workers and one scheduling coordinator. The scheduling coordinator is responsible for managing the allocation of visits and citizen care among the HHC-workers. Moreover, each team is supported by a group of nurses and needs assessors. The nurses are often located geographically close to the HHC-workers, and available for easy contact if needed. The needs assessors are often located in another geographic location. Therefore, the communication between HHC-workers and needs assessors primarily occurs in writing. In the self-governing teams, one team manager is responsible for 2–3 groups which each include one nurse and a maximum of 12 HHC-workers. Each group is responsible for planning their own work schedules on a daily basis. The scheduling coordinator assists all groups in each team with the planning of work schedules. Each team has a weekly inter-disciplinary meeting that includes HHC-workers, the nurses and a needs assessor.

### Data collection

**Fig. 2:**
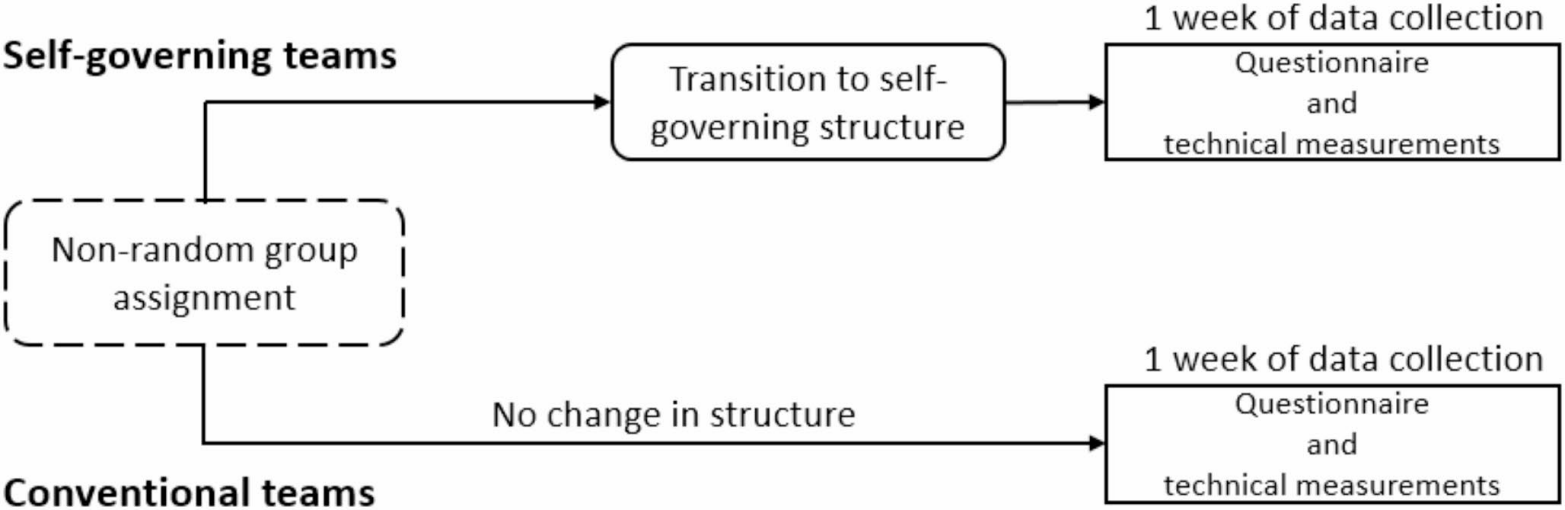
Timeline of activities in the self-governing and conventional teams, respectively

The study was performed as a natural experiment with a cross-sectional evaluation (Fig. 2). The data collection was performed between August 2023 and January 2024 and occurred over one working week per team (Monday-Friday). The data collection consisted of quantitative data comprising an electronic questionnaire and technical measurements of physical behaviour. All data were stored and analyzed according to the current guidelines for data protection (GDPR.EU, 2021).

#### Start-up meeting

The interactions with both self-governing and conventional teams were initiated with a start-up meeting between the HHC-workers and a member from our research group. During the start-up meeting, the HHC-workers were introduced to the components of the study through a PowerPoint presentation presented by the researcher. Moreover, each HHC-worker was given an envelope containing information about the project, a data processing agreement, an accelerometer and a guide explaining how to attach the accelerometer.

#### Outcomes

The primary outcome of this study was to compare LBP intensity in self-governing and conventional team structure. Secondary outcomes included (i) LBP duration and work limitations, (ii) neck/shoulder pain intensity, duration and work limitations, (iii) stress, (iv) productivity, (v) influence at work, (vi) meaning at work, (vii) sickness absence, (viii) interpersonal collaboration, and (ix) variation in physical behaviour.

On day one of data collection (Monday), HHC-workers received a unique link to a digital questionnaire via a text message to their mobile phone (Survey Xact, sourced from Ramboll Management Consulting, Aarhus, Denmark). The questionnaire consisted of questions regarding sociodemographic factors (i.e., age, gender, ethnicity, seniority, job title), perceived influence at work, measured on a 0-100 scale (Clausen et al. 2019), meaning at work, measured on a 0-100 scale (Clausen et al. 2019), interpersonal relationships (i.e. collaboration with colleagues, manager and needs assessors), measured on a 0-100 scale (Clausen et al. 2019), well-being, measured on a 0-100 scale (Topp et al. 2015), burn out, measured on a 0–8 scale (“COPSOQ II. The scales of the SHORT COPSOQ II questionnaire”, 2007), need for recovery, measured on a 0–4 scale from “never” to “always“ (Stevens et al. 2019), physical exertion, measured on a 0–10 scale (Borg, 1982), LBP intensity, measured on a 11-point Likert scale (Kuorinka et al. 1987), duration, measured with 0–28 days/month, and work limitations (yes or no), shoulder/neck pain intensity, measured on a 11-point scale (Kuorinka et al. 1987), duration, measured with 0–28 days/month, and work limitations (yes or no), stress, measured on a 5-point Likert scale from “never” to “always” (Clausen et al. 2019; Eskildsen et al. 2015), productivity, measured on a 11-point Likert scale from 0–10 (Kessler et al. 2003), and sickness absence, measured with 0–28 days/month (Burdorf et al. 1996). The HHC-workers in the self-governing teams in addition received questions concerning appraisal of the self-governing structure (i.e. (i) to what extent do you want to continue with being organised as self-governing? and (ii) do you think that other HHC-units could benefit from introducing self-governing teams? ).

The secondary outcome; variation in physical behaviour was measured using a triaxial accelerometer (SENS Motion^®^, Copenhagen, Denmark) attached to the right thigh midway on the line between the anterior inferior iliac spine and the top of the patella (Skotte et al. 2014) for five consecutive weekdays (Monday-Friday). The HHC-workers logged their work and sleep hours using an app. The accelerometer recorded, sampled and stored triaxial acceleration data at a frequency of 25 Hz with a measurement range of ± 4 g. The data was processed using Motus algorithm (“ActiMotus [Computer software],” 2024), to determine physical behaviour, including time spent sitting, standing still, standing with movement, slow walking (< 100 steps per minutes), fast walking (≥ 100 steps per minutes), stair climbing, running and cycling. These physical activities were then merged into three categories: (i) sitting (sitting and/or lying), (ii) standing (standing still and standing with movements), and (iii) active (slow walking, fast walking, stair climbing, cycling and running). Additionally, we also calculated cycling separately to gain insight into the extent to which HHC-workers utilized cycling for transportation between citizens. Non-movement periods lasting for more than 60 min were considered as non-wear time or sleep. Workdays with <4 h of accelerometer recordings were excluded from the analysis.

### Power calculation

A power calculation was performed to assess the statistical power of a t-test comparing mean LBP intensity scores (primary outcome) between self-governing teams and conventional teams. Based on a previous study (Korshøj et al. 2018) we aimed for a difference between the two groups of 1.2 in LBP intensity (on a 0–10 NRS). The standard deviation (SD) was estimated to be 2.3, derived from data obtained in a previous trial involving HHC-workers (Stevens et al. 2022). With a power of 0.80, and a significance level of 0.05 and an intra-cluster correlation coefficient of 0.05, a minimum of 58 participants within each group was required to achieve the desired statistical power.

### Statistical analyses

Descriptive statistics were conducted and summarized in group means, standard deviations and percentages across the teams. T-tests were used to compare outcomes between the two groups and the significance level was set to *p* ≤ 0.05. We generated violin plots to illustrate the distribution of average worktime in sedentary, standing and active physical behaviours per HHC-workers within self-governing and conventional teams, respectively. Moreover, we generated a violin plot illustrating the distribution of cycling during active worktime. All analysis were conducted in R version 4.1.3 (a language and environment for statistical computing).

## Results

**Fig. 3:**
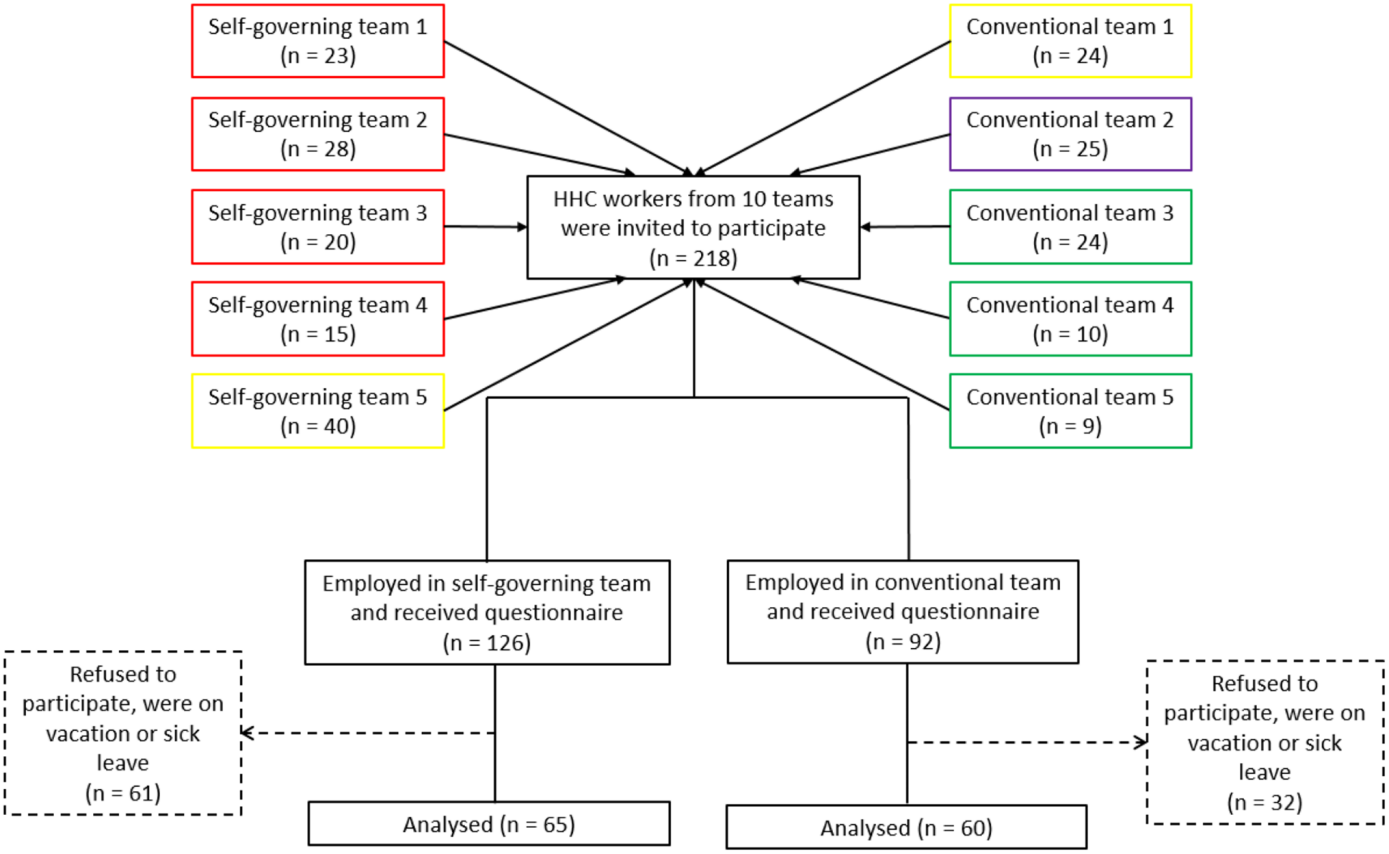
CONSORT flow diagram. Participants flow, leading to the final sample of 65 HHC-workers included in the measurements of LBP intensity (primary outcome) in the self-governing teams and 60 HHC-workers in the conventional teams. The colours represent the four municipalities from which the teams came from; red for Municipality 1, yellow for Municipality 2, purple for Municipality 3, green: Municipality 4

Figure 3 shows the 10 teams, consisting of five self-governing and five conventional teams from four municipalities that agreed to participate. Municipality 1 (coloured red in the flow diagram) contributed four self-governing teams; Municipality 2 (coloured yellow in the flow diagram) contributed one self-governing team and one conventional team; Municipality 3 (coloured purple in the flow diagram) contributed one conventional team; Municipality 4 (coloured green in the flow diagram) contributed three conventional teams. The 10 teams included a total of 218 HHC-workers (self-governing *n* = 126; conventional *n* = 92). 61 HHC-workers from self-governing teams and 32 HHC-workers from conventional teams did not want to participate, were on vacation or sick leave. The questionnaire was answered by 125 of the HHC-workers (self-governing *n* = 65; conventional *n* = 60). Answers from the 125 HHC-workers were all included in the analysis of the primary outcome (i.e. LBP intensity).

In addition, 55 of the HHC-workers (self-governing *n* = 38; conventional *n* = 17) participated in the technical measurements of physical behaviour. All 55 HHC-workers had ≥ 4 h of accelerometer recordings and were included in the statistical analysis of physical behaviour.

Table 1 shows the demographic breakdown of the HHC-workers included in the statistical analysis of the primary outcome, separated into self-governing and conventional teams. HHC-workers within the two team structures were similar.

**Table 1:** Demographic of the HHC-workers separated into self-governing and conventional teams

	Self-governing teams (*n* = 65)		Conventional teams (*n* = 60)	
	*N* (%)	Mean (SD)	*N* (%)	Mean (SD)
**Gender (female)**	59 (90.8)		52 (86.7)	
**Age (years)**		45.6 (11.6)		47.1 (11.2)
**Job title**				
HHC helper	35 (53.8)		34 (56.7)	
HHC assistance	22 (33.8)		22 (36.7)	
Other*	8 (12.3)		4 (6.7)	
**Seniority at current workplace**				
< 3 months	5 (7.7)		2 (3.3)	
3 months - <1 year	9 (13.8)		6 (10.0)	
1 year - <3 years	10 (15.4)		11 (18.3)	
3 years - <5 years	6 (9.2)		7 (11.7)	
5 years - <10 years	14 (21.5)		12 (20.3)	
≥ 10 years	21 (32.3)		22 (36.7)	
**Time in main occupation (hours/week)**		34.8 (2.7)		35.4 (3.0)

Values are presented as frequencies (N), percentages (%), and mean with standard deviations (SD). A statistical significance level was set to P-value ≤ 0.05. *Other; trainees and helpers without education

### Differences between HHC-workers in self-governing and conventional teams

Table 2 shows differences in health- and working environment factors between self-governing and conventional teams. The analysis showed no statistically significant differences in mean LBP intensity between HHC-workers in the self-governing teams and conventional teams. However, HHC-workers in the self-governing teams reported significantly higher levels of meaning at work (5.8 points on a 0-100 scale), as well as improved collaboration with manager (7.5 points on a 0-100 scale) and collaboration with needs assessors (11.9 points on a 0-100 scale) compared to HHC-workers in conventional teams.

**Table 2:** Differences in outcomes between self-governing and conventional teams as measured by questionnaire

	Self-governing teams (*n* = 65)	Conventional teams (*n* = 60)	Differences in means between teams	Significance level between teams
	Mean (SD)	Mean (SD)	MD	*P*-value
**Low back pain**				
Intensity (0–10) *	4.1 (2.9)	4.0 (2.9)	0.1	0.74
Duration (0–28 days) **	7.5 (9.6)	7.2 (9.6)	0.3	0.86
Limitation (% yes) **	19 (39.6)	22 (47.8)	3	0.42
**Neck/shoulder pain** ^**^				
Intensity (0–10)	3.5 (3.0)	2.8 (2.9)	0.7	0.20
Duration (0–28 days)	6.6 (9.6)	5.6 (9.0)	1.0	0.56
Limitation (% yes)	15 (34.9)	15 (42.9)	0	0.47
**Stress** ^**^				
The last two weeks (0-100)	43.8 (25.4)	40.4 (21.6)	3.4	0.42
Perceived stress (0–16)	5.9 (2.3)	5.5 (2.6)	0.4	0.37
Productivity^**^ (0–10)	8.2 (2.0)	8.2 (1.7)	0	0.93
Influence at work** (0-100)	68.2 (15.9)	66.8 (17.5)	1.4	0.64
Meaning at work^**^ (0-100)	77.3 (16.7)	71.5 (17.0)	5.8	0.05
Sickness absence^**^ (0–28 days)	2.0 (4.6)	2.1 (3.9)	0.1	0.85
**Interpersonal** collaboration** (0-100)				
Collaboration with colleagues	72.7 (21.6)	68.0 (19.7)	4.7	0.21
Collaboration with manager	74.9 (20.4)	67.4 (18.9)	7.5	0.04
Collaboration with needs assessors	61.4 (18.0)	49.5 (15.2)	11.9	<0.01
**Well-being** (0-100)	65.5 (17.9)	61.6 (20.6)	3.9	0.21
**Burn out** (0–8)	3.9 (1.7)	4.0 (1.6)	0.1	0.76
**Need for recovery** (0–4)	1.9 (0.8)	2.0 (0.8)	0.1	0.47
**Physical exertion** (0–10)	5.8 (2.1)	6.0 (2.5)	0.2	0.71

Values are presented in means, standard deviation (SD) and mean difference (MD). A statistical significance level was set to P-value ≤ 0.05. ^*^ = Primary outcome, ^**^ = Secondary outcomes

**Fig. 4:**
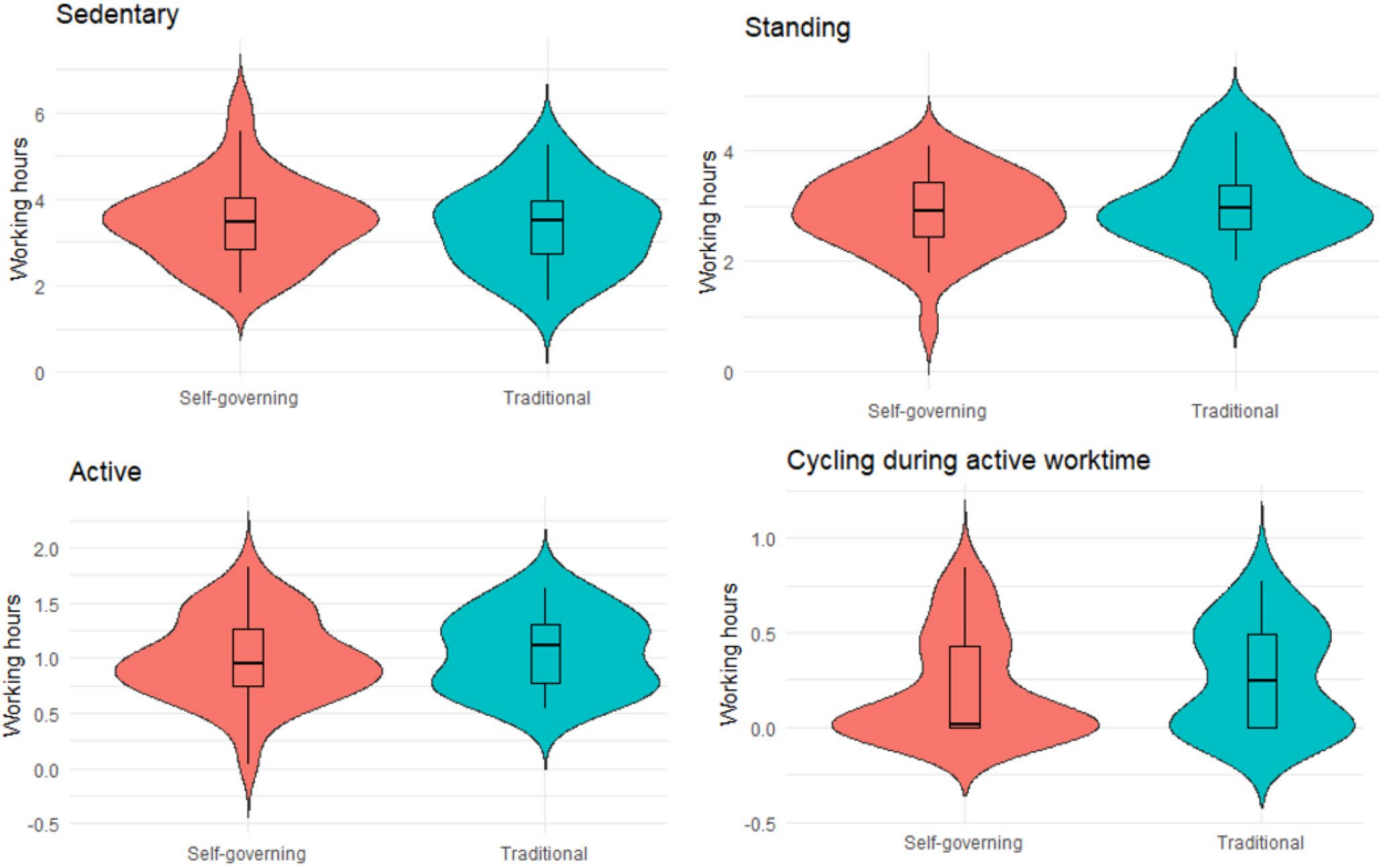
Average worktime per HHC-worker in hours spent sedentary, standing, active, and cycling. The violin plots depict information about the distribution of the data in self-governing teams and conventional team, respectively. The box displays the median, 25th and 75th percentile, and the black lines show the rest of the distribution

A total of 1500 working hours (self-governing = 1042 h; conventional = 458 h) measured with accelerometers were included in the analysis for the secondary outcome; variation in physical behaviours. This equated to an average of 3.7 working days measured per HHC-workers in the self-governing teams and 3.6 working days measured in conventional teams. The composition of physical activity during the workdays were divided into sedentary, standing and active. Additionally, active work time spent cycling was treated separately. Figure 4 illustrate that the variation in sedentary, standing, active and cycling during active worktime was somewhat similar between the two group structures. However, variation in sedentary, active and cycling during active worktime seems to be lower in self-governing teams compared to the conventional teams. Opposite, the variation in standing tends to be lower in conventional teams than in self-governing teams.

### Appraisal of self-governing teams

The majority (72.3%) of the HHC-workers within self-governing teams stated that they wanted to continue with being organized as self-governing to a high or very high degree. Additionally, the majority (72.3%) stated that other HHC-teams to some or very high degree could benefit of adopting a self-governing team structure (data not presented).

## Discussion

This study gives a novel contribution to our understanding of the differences in LBP and work-related factors in self-governing compared to conventional teams. No significant differences in LBP or neck/shoulder pain were found. However, significant differences in the working environment favoring the self-governing team structure were revealed, including meaning at work, collaboration with manager and collaboration with needs assessors.

The observed improvements in meaning at work and interpersonal collaborations may be considered as early indicators of positive changes in the working environment within the self-governing teams. This aligns with previous studies that identifies associations between interpersonal relationships and job satisfaction (Zolak Poljašević et al. 2021), as well as between the sense of meaning at work and stress (Annison and Davidson 2023). Both job satisfaction and stress are well-established factors that influence musculoskeletal pain (Linton 2001; Vinstrup et al. 2020). Therefore, it is possible, that HHC-teams included in this study have not been self-governing for a sufficient length of time for the positive changes in meaning at work and interpersonal collaborations to translate into measurable changes in health outcomes, including reduction in LBP intensity. To explore whether these factors contribute to a reduction in LBP intensity among HHC-workers in self-governing teams over time, we acknowledge that a longitudinal study would be a valuable approach for future research.

Other possible explanations for the lack of difference in LBP intensity could be that the self-governing team structure does not impact the amount of physical workload, as the work tasks remain the same regardless of the team structure. Nor does the self-governing structure influence how the HHC-workers perform their physically demanding work tasks. However, as it is embedded in the self-governing structure that each group is responsible of planning their daily work schedules, this could lead to a more equal distribution of physically demanding work tasks among HHC-workers. This has previously been suggested as a beneficial organizational approach to prevent LBP among eldercare workers (Wester et al. 2025). On the other hand, there is a possibility that some HHC-workers in the self-governing teams will secure themselves more favourable schedules, resulting in an even more unequal distribution of the physically demanding work tasks, affecting musculoskeletal pain negatively.

We calculated the average distribution of daily working time spent in sedentary, standing, and active physical behaviours, revealing that the variation was somewhat similar in self-governing teams compared to the conventional teams. Moreover, the average distribution of physical behaviours at work in both the self-governing and conventional teams is consistent with previous accelerometer measurements of physical behaviours among HHC-workers (Tjøsvoll et al. 2022). The consistency in our findings was expected, as the job tasks remain similar regardless of the organizational structure of the HHC-teams. In addition to the initial calculations, we calculated the amount of time spent cycling during active working hours, as the mode of transportation between citizens must be assumed to influence the level of active work time. Our findings indicated that HHC-workers in conventional teams spent more of their working time cycling. This could be due to the geographical areas that each teams covers, though we have no data to confirm this. The limited participation in the accelerometry measurements prevented us from assessing the inter-individual physical behaviour between team members in self-governing teams compared to HHC-workers in conventional teams. We recommend that further studies consider exploring this aspect further, as it could provide valuable insight into the distribution of work tasks among HHC-workers in the self-governing structure.

The significantly higher level of perceived meaning at work among HHC-workers in self-governing teams (5.8 points on a 0-100 scale) compared to HHC-workers in conventional teams, is likely attributed to the high degree of autonomy in their work, and this has likely contributed to their sense of competence. Previous studies have shown that having sufficient influence to engage with work tasks in a relevant manner benefits employees’ job satisfaction and their experience of meaning (Andersen et al. 2022; Sasser and Sørensen 2016; Semmer et al. 2019). Furthermore, the significantly higher level of collaboration with their manager (7.5 points on a 0-100 scale) and needs assessors (11.9 points on a 0-100 scale) compared to those in conventional teams, may likely be due to the regular interdisciplinary meetings that replaced the written communication. This change may foster an interdisciplinary collaboration that may positively impact the workers’ job satisfaction (Foà et al. 2020; Ylitörmänen 2021). Additionally, the establishment of a faster communication pathway will allow workers to complete their work tasks more efficient, which, in turn, is expected to enhance their sense of meaning at work (Andersen et al. 2022; Sasser and Sørensen 2016; Semmer et al. 2019).

Our study provides novel insights into the differences in the work environment of HHC-workers in self-governing versus conventional team structures. The absence of significant differences in the musculoskeletal pain outcomes emphasizes the continued necessity of developing interventions targeting musculoskeletal pain and health among HHC-workers.

The self-governing team structure does not have a structural focus on employees’ health. However, we see a clear opportunity to integrate such focus into the self-governing structure, as factors like high autonomy and flexibility in task management provide a chance for the HHC-workers to continuously consider their health when distributing work tasks.

A particularly well-aligned approach for such an integration is the Goldilocks Work principle, which aims to organize productive work in a way that is ‘just right’ for the workers’ health (Holtermann et al. 2019; Straker et al. 2018). The Goldilocks Work approach has previously been developed and tested in the context of HHC (Lohne et al. 2025; Vilhelmsen et al. Submitted). However, one of the studies focused solely on the physical aspects of the work tasks (Lohne et al. 2025), while neither of the studies were specifically developed to address the self-governing structure. Furthermore, both studies found that their interventions were not adequately implemented within the organization, highlighting the need for further research with a strong focus on the developing and implementation of interventions in HHC.

Thus, we argue that a logical next step could be to integrate a Goldilocks Work intervention in the self-governing team structure, using a participatory approach, as a promising way to reduce musculoskeletal pain and increase health among HHC-workers. The development and implementation of such an intervention would provide the HHC-workers with the skills to distribute a more appropriate and healthy distribution of work tasks, which could potentially reduce musculoskeletal pain and increase health.

### Strengths and limitations

The natural experiment design is a methodological strength since it provides insight into the potential effect of implementation of self-governing teams in a realistic context. However, the use of a natural experiment design could be seen as a limitation, as natural experiments are more susceptible to bias (Craig et al. 2012, 2022). Due to practical matters, we were not able to collect baseline data as the self-governing teams had already restructured when it was possible to start the data collection. Consequently, we cannot ensure that the observed differences between the two team structures are solely attributable to the transition into the self-governing structure, as other unmeasured factors may have influenced the outcomes. However, rather than being a limitation, we argue that the design is a strength in the study, as the natural experiment design allows us a more realistic understanding of how implementation of the self-governing team structure may impact the working environment among HHC-workers. We did our data collection, when it was still possible to have a comparison group of teams, who had not yet undergone a restructure into self-governing teams, which is not always available in natural experiments.

Possible biases should be noted when interpreting the results of our study. The teams that applied for funding resources for implementing the self-governing team structure may already have been well-functioning, with strong collaboration skills across professions. They therefore may have a higher capacity to experiment with the new organizational structure. Moreover, the voluntary recruitment process could have introduced a selection bias. We cannot determine if only well-functioning HHC-teams chose to participate, which means our results may not be representative for all HHC-teams across Denmark.

## Conclusion

Our study provides novel insights and enhances the understanding of musculoskeletal pain as well as work-related factors among HHC-workers in conventional and self-governing team structures. While no significant difference in musculoskeletal pain was found, self-governing teams demonstrated significant benefits in terms of increased meaning at work and improved collaboration with manager and needs assessors. Moreover, the HHC-workers in self-governing teams reported high appraisal of the self-governing structure. The similar level of LBP and shoulder/neck pain highlights the need for more comprehensive and tailored efforts to improve the physical health of HHC-workers. Future research should explore additional strategies to address these health issues among HHC-workers within the self-governing team structure.

## Data Availability

Raw data are not public available to preserve individuals’ privacy.
